# Fatigue Experiment Study on Internal Force Redistribution in the Negative Moment Zone of Steel–Concrete Continuous Composite Box Beams

**DOI:** 10.3390/ma16072927

**Published:** 2023-04-06

**Authors:** Yongzhi Gong, Qi Zhong, Yingjie Shan, Yu Sun

**Affiliations:** 1 School of Civil Engineering, Central South University, Changsha 410083, China; 2 China Railway SIYUAN Survey and Design Group Co., Ltd., Wuhan 430063, China

**Keywords:** steel–concrete continuous composite box beam, fatigue performance, negative moment zone, internal force redistribution

## Abstract

Due to the accumulated fatigue damage in steel–concrete continuous composite box beams, a plastic hinge forms in the negative moment zone, leading to significant internal force redistribution. To investigate the internal force redistribution in the negative moment zone and confirm structural safety under fatigue loading, experimental tests were conducted on nine steel–concrete continuous composite box beams: eight of them under fatigue testing, one of them under static testing. The test results showed that the moment modification coefficient at the middle support increases during the fatigue process. When approaching fatigue failure, an increase of 1.0% in the reinforcement ratio or 0.27% in the stirrup ratio results in a reduction of 13% in the moment modification coefficient. Furthermore, a quadratic function model was proposed to calculate the moment modification coefficient of a steel–concrete continuous composite box beam during the fatigue process, which exhibited good agreement with the experimental results. Finally, we verified the applicability of the plastic hinge rotation theory for steel–concrete continuous composite box beams under fatigue loading.

## 1. Introduction

Compared with ordinary reinforced concrete beams, steel–concrete composite beams exhibit the advantages of light weight, small cross-section, good ductility, and high seismic resistance [1,2,3]. Since the 20th century, steel–concrete composite beams have been widely used in bridge structures [4,5]. In this engineering background, fatigue damage is one of the most important forms of damage to steel–concrete composite beams, as various kinds of damage to composite beams can occur during the fatigue process.

In recent research, many scholars have conducted fatigue tests and investigated the fatigue failure mechanisms in steel–concrete composite beams. The results indicate that under fatigue loading, steel–concrete continuous composite beams experience stud failure and concrete cracking, leading to degradation decrease in stiffness. Lin et al. [6] conducted fatigue tests on two steel–concrete composite beams. The test results confirmed the failure of the bonds between the studs and concrete during the fatigue process. Li et al. [7] divided the fatigue damage of the steel–concrete composite beams into three stages. Zhou et al. [8] conducted fatigue tests on a full-scale model of a steel–concrete joint section of a hybrid girder cable-stayed bridge. The test results showed that after 3 million load cycles, the shear studs near the loading end entered a plastic state, and the overall rigidity decreased. Wang et al. [9] proposed a calculation method for the deflection of steel–concrete composite beams under fatigue loading. Liang et al. [10] conducted fatigue tests on 11 push-out specimens of stud connectors and improved a “fast-slow-fast” three stage load–slip model. Wang et al. [11] investigated the correlations among the stiffness degradation, residual deflection, and relative slip growth during the fatigue test. Based on the test results, a calculation model for the residual stiffness of composite beams in response to fatigue loading cycles was proposed. Therefore, the cumulative damage can significantly affect the fatigue behavior and structural safety of steel–concrete continuous composite box beams. Moreover, many experimental studies have shown that parameters such as the reinforcement ratio, stirrup ratio, and shear connection degree have a significant influence on the fatigue performance of such composite beams [12,13,14,15,16].

In addition, in continuous composite beams, the negative moment at the middle support leads to an unfavorable situation where the concrete slab is under tension and the steel beam is under compression [17]. When there are sufficient concrete cracks and stiffness degradation, a plastic hinge forms at the middle support, leading to an internal force redistribution in the continuous beams [18,19]. To date, many researchers have investigated the internal force redistribution of steel–concrete composite continuous beams under static tests. Gao et al. [20] carried out fatigue tests on two double-layer composite beams, and the results indicated that the degradation of bond-slip between the concrete and reinforcement in the negative moment zone caused the degradation of stiffness and the development of cracks. Wu et al. [21] studied the feasibility of improving the crack resistance of composite beams by releasing the interfacial slip effect within the negative bending moment zone. Zhang et al. [22] studied the redistribution of the internal force of a steel–concrete composite continuous box beam and found that the influence of the slip at the middle support should be fully considered. Su et al. [23] studied the redistribution of shear stress under prestressing force in the negative moment zone. Hu et al. [24] conducted static tests on three prestressed steel–concrete continuous composite beams; the results indicated that the main factors in the redistribution of internal forces are the formation of plastic hinges, cracking of concrete slabs, and failure of shear connectors. Sun [25] proposed a method of calculating the moment modification coefficient based on the plastic hinge rotation theory.

However, few studies have been carried out on the internal force redistribution of steel–concrete continuous composite beams in the negative moment zone under fatigue tests. Previous studies [26,27] have shown that fatigue damage and the internal force redistribution at the middle support increase with the number of fatigue cycles, but the development law remains unclear. Hence, it is necessary to propose a model that can predict the internal force redistribution throughout the entire fatigue process. Moreover, it remains to be verified whether the plastic hinge rotation theory is applicable to steel–concrete continuous composite box beams under fatigue loading.

To investigate the internal force redistribution condition of steel–concrete continuous composite box beams during the fatigue loading process, eight specimens (FSCB-1 to FSCB-8) with different parameters—including load amplitude, reinforcement ratio, stirrup ratio, and shear connection degree—were subjected to fatigue load testing. One specimen (FSCB-0) was subjected to static load testing. Based on the experimental results, a moment modification coefficient–fatigue process model was proposed, showing good agreement with all specimens. Furthermore, by comparing the calculated results with the experimental results, we verified the applicability of plastic hinge rotation theory for steel–concrete continuous composite box beams under fatigue loading.

## 2. Experimental Test

### 2.1. Design of the Test Specimens

**Figure 1:**
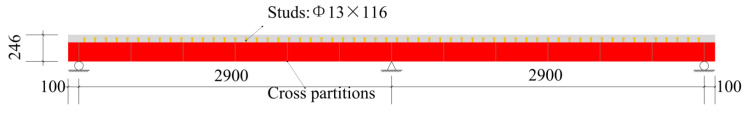
Longitudinal section of specimen.

**Figure 2:**
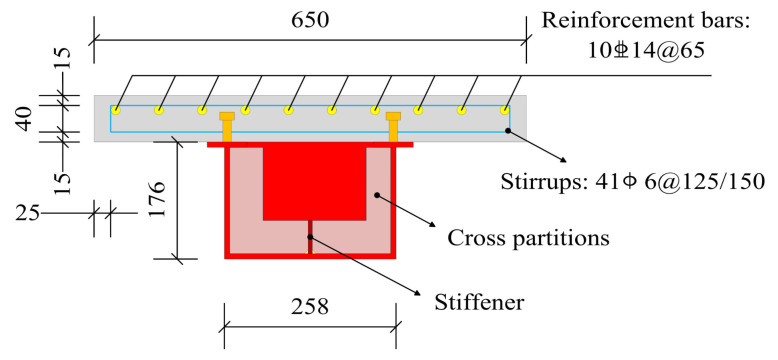
Cross section of specimen.

Based on the experiment of Zhang et al. [22], nine 1:8-scaled models of continuous composite box beams were fabricated according to the Chinese code (JTG 3362-2018) [28]. FSCB-0 was used for the static comparison test, and FSCB-1 to FSCB-8 were used for the fatigue tests. Each specimen was 6000 mm long, with 2900 mm intervals between adjacent supports. The steel box was welded from 8 mm Q235 steel plates using semi-automatic CO_2_ gas-shielded welding. The fillet weld joints had a leg size of 6 mm. Thirteen cross partitions of 8 mm thickness were installed in the steel box, and a 50 mm × 6 mm longitudinal stiffening rib was installed at the bottom of the steel box to prevent shear failure before bending failure occurred. The steel box had a width of 258 mm and a height of 176 mm, with the upper flange width being 60 mm. The concrete slab was cast with C30 concrete, with a width of 650 mm and a height of 70 mm. The 6 mm diameter HPB300 rebars were used as the stirrups, and the 14 mm diameter HRB400 rebars were used as the reinforcement steel. The concrete slab and steel box were connected by 13 mm diameter and 50 mm high studs. The size of each component and the layout of the reinforcements and studs are shown in Figure 1 and Figure 2. The parameters considered in this test were the load amplitude, stirrup ratio, longitudinal reinforcement ratio, and shear connection degree. The design details of the specimens are shown in Table 1. The material properties of the concrete and steel used in this test are shown in Table 2 and Table 3, respectively.

### 2.2. Instrumentation

**Figure 3:**
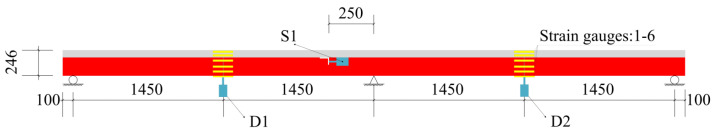
Arrangements of strain gauges and dial gauges.

The arrangement of the measurement points for the tests is shown in Figure 3. A spoke-type pressure sensor was used to measure the load at each support of the specimen beam. Additionally, two displacement transducers (D1 and D2) were arranged at each mid-span position of the continuous composite box beam to measure vertical displacement. At a distance of 25 cm from the middle support, a displacement transducer (S1) was fixed to the side of the steel box, and an L-shaped steel plate was fixed at the bottom of the concrete slab. The slippage between the steel box and the concrete slab during the test was measured by recording the relative displacement between S1 and the L-shaped steel plate.

To verify whether the strain along the height of the section during fatigue loading satisfied the plane section assumption, six strain gauges were arranged at each mid-span position along the height of the specimens. The strain gauges were numbered from 1 to 6, from the top of the concrete slab to the bottom of the steel box. Two strain gauges were arranged along the concrete slab’s thickness, which were 10 mm away from the top and bottom of the slab. Three strain gauges were arranged on the steel box web along the height direction, and two strain gauges were arranged on the bottom of the steel box’s lower flange—one near the center and one near the edge.

### 2.3. Experimental Program

**Figure 4:**
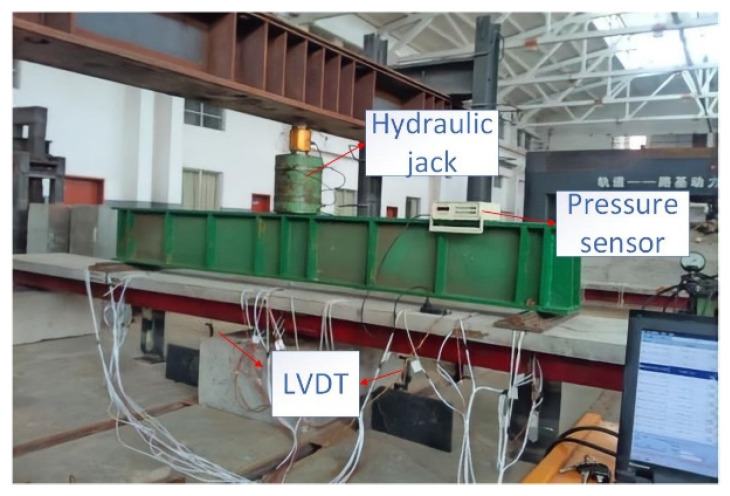
Schematic view of the fatigue tests.

**Figure 5:**
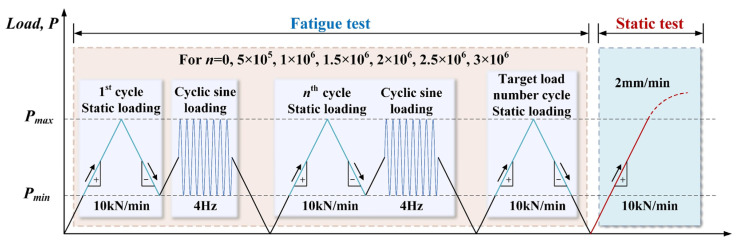
Loading procedure for the fatigue tests.

The test was conducted in the National Engineering Laboratory of High-speed Railway Construction Technology at Central South University. A two-point symmetric loading method was used, and the load was uniformly transferred to the loading point by a PMS-500 hydraulic jack through the spread beam, as shown in Figure 4. In accordance with the Chinese code (GB/T 50152-2012, 2012) [29], the loading frequency was set to 4 Hz under sinusoidal pulsating loading, and the fatigue load details for specimens FSCB-1 to FSCB-8 are depicted in Figure 5. Before the fatigue load test, the preload was cast on the specimens to check the loading condition and to make sure all measurements worked normally. During the fatigue load procedure, the fatigue load was stopped when the number of fatigue load cycles reached 0, 5 × 10^5^, 1 × 10^6^, 1.5 × 10^6^, 2 × 10^6^, 2.5 × 10^6^, and 3 × 10^6^, in order to conduct the intermediate static tests. The fatigue life of each specimen was determined based on the longest fatigue life of the steel material. Specifically, when the first crack could be observed on the steel box, the number of cycles at this point was considered to represent the fatigue life of the steel–concrete continuous composite box beam [30]. The fatigue test was terminated when the crack width of the steel box reached 5 mm. Eventually, a final static test was conducted on FSCB-1 to FSCB-8 to determine the residual load capacity after fatigue failure.

## 3. Results and Discussion

### 3.1. Damage Mode and Fatigue Life

Specimen FSCB-0 was subjected to a static load, and observable damage occurred as the load increased. At 85 kN, the first crack appeared in the top surface of the concrete slab at the middle support. At 382 kN, with a loud sound, the slippage between the steel box and the concrete slab began to increase, and some studs near the middle support were sheared off. Finally, at 820 kN, the concrete near the mid-span was crushed; however, no cracks were observed on the steel box.

For specimens FSCB-1 to FSCB-8, which were subjected to fatigue loads, the same damage characteristics appeared sequentially as the fatigue load cycle (defined as *N_c_*) increased. Taking FSCB-1 as an example, when *N_c_* = 0, a few cracks appeared on the top surface of the concrete slab at the middle support. When *N_c_* = 1 × 10^4^, the width of initial cracks on the concrete slab at the middle support started to expand. When *N_c_* = 1 × 10^6^, the cracks in the concrete slab at the middle support gradually expanded from the edges to the middle until they penetrated the top surface of the concrete slab. When *N_c_* = 1.3 × 10^6^, obvious separation could be observed between the steel box and the concrete slab, and fatigue shear failure occurred at the bottom section of some studs near the middle support. When *N_c_* = 1.5 × 10^6^, a few cracks appeared on the bottom surface of the concrete slab at the mid-span and gradually developed toward the top surface. When *N_c_* = 1.92 × 10^6^, the first fatigue crack on the upper flange of the steel box at the middle support could be observed with the naked eye. Fatigue loading continued until *N_c_* = 2.01 × 10^6^, at which point the width of the crack on the steel box had expanded to 5 mm. The fatigue test was then terminated, and the final static test was conducted. In the final static test, the crack on the steel box vertically penetrated the whole steel box section from the upper flange to the bottom of the web, while the lower flange of the steel box remained intact. The length of the concrete slab’s crack distribution area reached 1.15 m. The development of concrete cracks is shown in Table 4.

**Figure 6:**
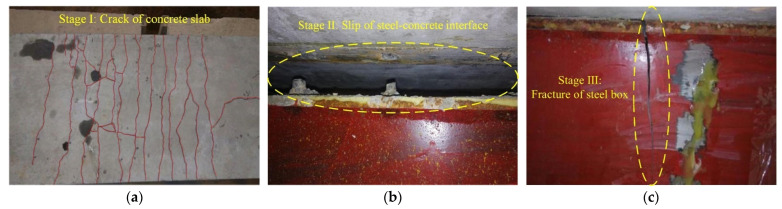
Schematic diagram of each stage under fatigue tests: (**a**) stage I; (**b**) stage II; (**c**) stage III.

According to the fatigue test phenomena, the fatigue damage of the continuous composite box beam can be divided into three stages: (1) Stage I: in the early stage of fatigue loading, concrete cracks appeared at the middle support and expanded with increasing fatigue load cycles. (2) Stage II: as the number of fatigue load cycles increased, some studs near the middle support were sheared off, and obvious separation and slippage between the concrete slab and the steel box could be observed on both sides. (3) Stage III: the continuous composite box beam failed to bear the load as a single unit, and cracks appeared on the upper flange of the steel box and expanded downward to the middle support with increasing fatigue load cycles. After the upper flange of the steel box fractured, the continuous composite box beam could be considered to have undergone fatigue failure, and the number of fatigue load cycles at this time could be taken as the fatigue life of the beam (defined as *N_i_*). The three-stage damage characteristics of the continuous composite box beam are shown in Figure 6. The residual load capacity in the final static test, the fatigue life, and the corresponding fatigue load cycles of each fatigue stage are shown in Table 5.

**Figure 7:**
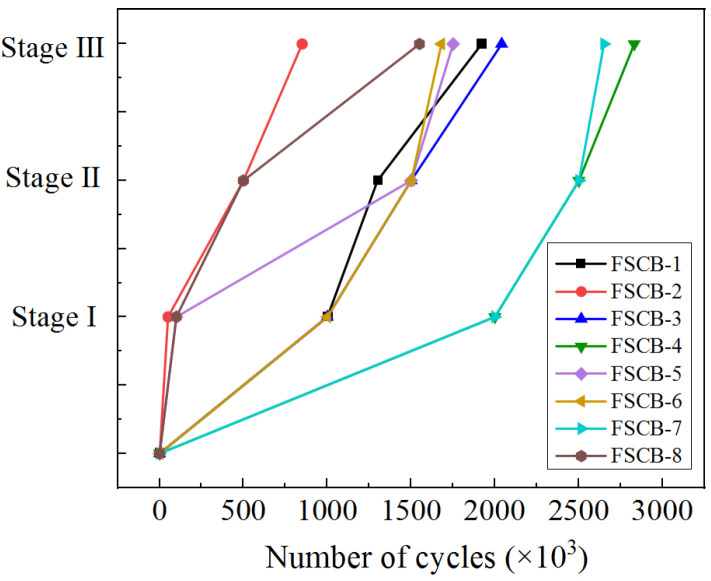
Order of fatigue damage occurrence.

**Figure 8:**
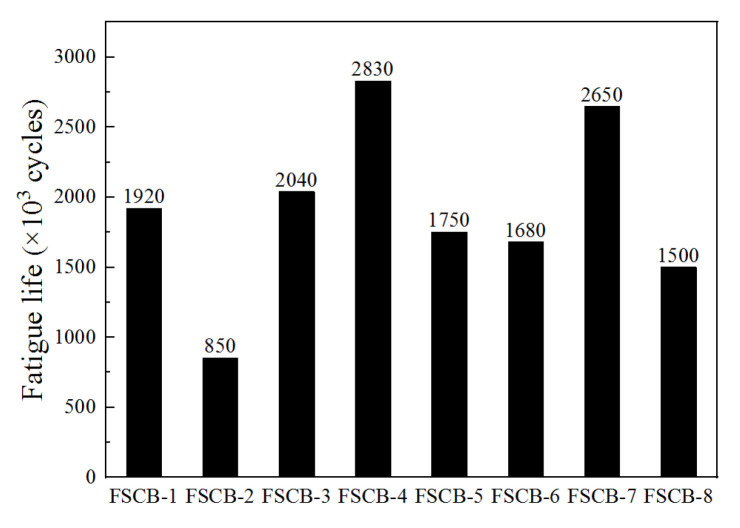
Fatigue life of specimens.

Figure 7 and Figure 8 show the occurrence of fatigue damage and the fatigue life of specimens FSCB-1 to FSCB-8, respectively. Compared with FSCB-1, the steel–concrete interface separated earlier in FSCB-2 and FSCB-8, and the fatigue life decreased by 55.7% and 21.9%, respectively. In contrast, FSCB-4 and FSCB-7 experienced fatigue damage much later than FSCB-1, and the fatigue life increased by 47.4% and 38.0% compared to FSCB-1, respectively. According to Figure 4, FSCB-2, FSCB-5, FSCB-6, and FSCB-8 exhibited a 170–350 mm increase in the crack distribution area on the concrete slab in the negative moment zone, compared to FSCB-1. This indicates that the larger load, lower reinforcement ratio, stirrup ratio, and shear connection degree accelerate the development of cracks in the negative moment zone under fatigue loading, thereby hastening the overall failure process of the beam. In addition, under actual loading conditions, the fatigue life of steel–concrete continuous composite box beams will decrease with the increase in specimen size, due to the influence of material defects [9,31].

### 3.2. Plane Section Assumption

**Figure 9:**
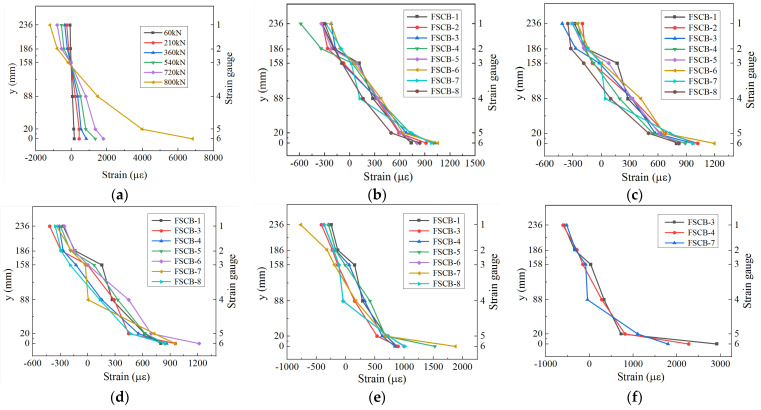
The distribution of strain along the height of the cross-section in the mid-span: (**a**) FSCB-0; (**b**) *N_c_* = 0; (**c**) *N_c_* = 5.0 × 10^5^; (**d**) *N_c_* = 1.0 × 10^6^; (**e**) *N_c_* = 1.5 × 10^6^; (**f**) *N_c_* = 2.0 × 10^6^.

The distribution of strain along the height of the cross-section in the mid-span of FSCB-0 to FSCB-8 is shown in Figure 9. The curves show that the cross-sectional strain of FSCB-0 was approximately linearly distributed along the height of the beam in the early stage of test loading, which is consistent with the plane section assumption. When loaded to 800 kN, the average slope value of the y-strain curve for strain gauges 1–5 was 0.042, and the slope value of the y-strain curve for strain gauges 5–6 was 0.007.

During the early stages of fatigue loading, the y-strain curves for FSCB-1 to FSCB-8 were similar and maintained linearity, conforming to the plane section assumption. However, when *N_c_* = 1.5 × 10^6^, FSCB-5 and FSCB-7 exhibited an average slope value of 0.222 and 0.149, respectively, for strain gauges 1–5, and a slope value of 0.024 and 0.017, respectively, for strain gauges 5–6. When *N_c_* = 2 × 10^6^, FSCB-3 and FSCB-4 had an average slope value of 0.164 and 0.152, respectively, for strain gauges 1–5, and a slope value of 0.018 and 0.014, respectively, for strain gauges 5–6. This indicates that at the end of the static loading process or fatigue loading process, the steel box yielded below the height of 22 mm, and the axial strain along the longitudinal direction did not obey the plane section assumption.

### 3.3. Slippage of Concrete–Steel Box Interface

**Figure 10:**
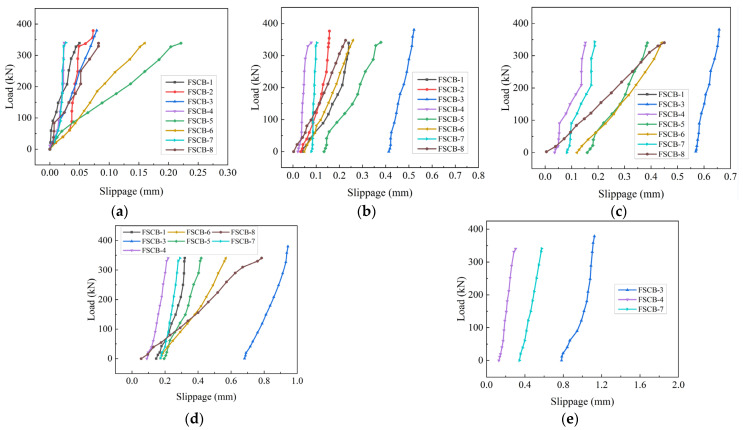
Load–slip curves at a distance of 25 cm from the middle support: (**a**) *N_c_* = 0; (**b**) *N_c_* = 5.0 × 10^5^; (**c**) *N_c_* = 1.0 × 10^6^; (**d**) *N_c_* = 1.5 × 10^6^; (**e**) *N_c_* = 2.0 × 10^6^.

After a certain number of fatigue load cycles, the load–slip curves at a distance of 25 cm from the middle support for the specimens before failure are shown in Figure 10. The slippage consisted of plastic slippage and elastic slippage, which were caused by the fatigue load and the static load, respectively. In the initial stages of the fatigue process, the ultimate load capacity of the studs exceeded the shear force caused by the upper load limit, leading to elastic deformation of the studs and the concrete. As the number of fatigue cycles increased, the bonds between the concrete slab and the studs gradually loosened, resulting in plastic slip at the interface. Eventually, fatigue shear failure occurred at the bottom section of some studs, causing a sudden increase in plastic slip. As the specimens approached fatigue failure, the average plastic slippage of FSCB-1 to FSCB-8 was 0.260 mm, while the elastic slippage of FSCB-0 at yield loading was 2.110 mm. This implies that the average plastic slippage accounted for 12.3% of the elastic slippage. Thus, it is crucial to consider plastic slippage due to fatigue load when detecting the slippage of steel–concrete continuous composite box beams.

### 3.4. Load–Deflection Response and Residual Stiffness Degradation

**Figure 11:**
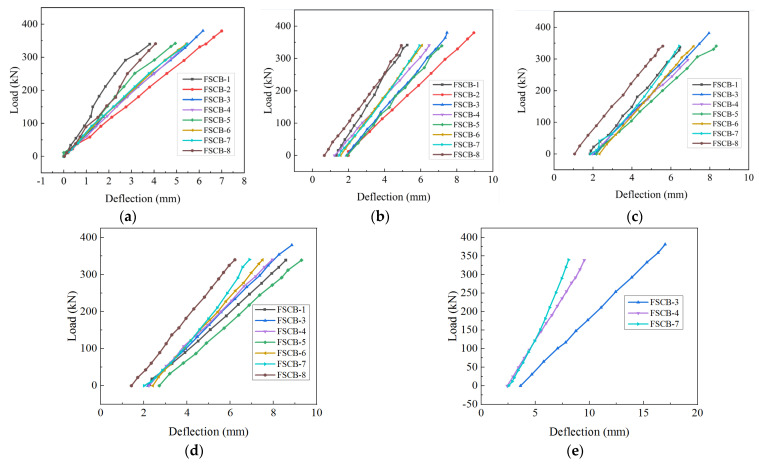
Load–deflection curves at the mid-span: (**a**) *N_c_* = 0; (**b**) *N_c_* = 5.0 × 10^5^; (**c**) *N_c_* = 1.0 × 10^6^; (**d**) *N_c_* = 1.5 × 10^6^; (**e**) *N_c_* = 2.0 × 10^6^.

Figure 11 shows the load–deflection curves at the mid-span of each continuous composite box beam when *N_c_* reached 0, 5 × 10^5^, 1 × 10^6^, 1.5 × 10^6^, and 2 × 10^6^. Within 2 × 10^6^ fatigue load cycles, the specimens were within the elastic range, and the load–deflection was essentially maintained as a linear relationship. As the number of fatigue load cycles increased, the stiffness gradually degraded and the residual deflection increased. According to a previous study by Huang et al. [30], the stiffness at the mid-span can be calculated using Equation (1), and the residual stiffness coefficient *R* can be defined using Equation (2):

*f* = *α* × *M* × *L*^2^/*B* (1)

*R* = *B_n_*/*B*_0_ (2)

where *f* is the deflection of the mid-span, *M* is the moment of the mid-span, *B* is the stiffness, *α* is the coefficient that takes account of the support and the load, *B_n_* is the stiffness when *N_c_* = *n*, and *B*_0_ is the stiffness when *N_c_* = 0.

**Figure 12:**
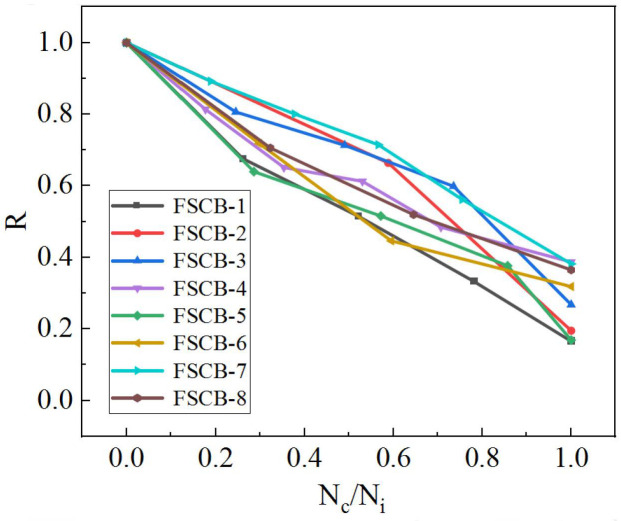
Stiffness degeneration curves.

The residual stiffness coefficient degeneration curves of FSCB-1 to FSCB-8 are shown in Figure 12; to make the test data with different parameters comparable, normalization was carried out (*N_c_/N_i_* indicates the process of fatigue failure). The results demonstrated that the residual stiffness coefficient of the continuous composite box beams decreased with increasing fatigue load cycles, following an “S-shaped” pattern with three stages: In the first stage, the residual stiffness coefficient decreased by about 20% to 30%, and the rate of decrease was stable; this stage accounted for about 20% of the fatigue life. In the second stage, the residual stiffness coefficient decreased slowly; this stage accounted for about 50% of the fatigue life. In the third stage, when approaching fatigue failure, the residual stiffness coefficient decreased rapidly and ultimately reached a value between 0.165 and 0.387. Among the eight continuous composite box beams tested, FSCB-4 and FSCB-7 exhibited the highest residual stiffness coefficients when approaching the end of their fatigue life, with values of 0.387 and 0.382, respectively. Overall, the residual stiffness coefficient curves were consistent with the three-stage damage characteristic, and the results indicated that continuous composite box beams with higher reinforcement or stirrup ratios experience a slower decrease in stiffness when approaching fatigue failure.

## 4. Analysis and Calculation of Internal Force Redistribution

### 4.1. Internal Force Redistribution at the Middle Support during the Fatigue Progress

Previous studies have shown that the formation of the plastic hinge will cause obvious internal force redistribution at the middle support of continuous composite box beams [20,21,22,23,24,25,26,27]. Table 6 shows the measured force (not including self-weight) at the middle support under the target number of loading cycles, with an applied static load of 340 kN. The test results show that the middle support force decreases with the increase in the number of fatigue load cycles, while the side support force increases with the increase in the number of fatigue load cycles. When approaching fatigue failure, the ratio of side support force to middle support force rapidly increases. According to the test results, this phenomenon can be attributed to the change in flexural stiffness and the effect of slippage. In the early stages of fatigue loading, the flexural stiffness of the beam is not significantly affected, and the load is distributed evenly among the supports. As the number of fatigue cycles increases, the cumulative fatigue damage results in the formation of the plastic hinge in the negative moment zone, leading to internal force redistribution in the beams.

The internal force redistribution at the middle support can be expressed by Equation (3) [27]:

*β_exp_* = (*M* − *M*′)/*M* (3)

*M*′ = (*F* − *F_m_*) × *l*/2 − *F* × *l*/4 (4)

where *β_exp_* is the experimental moment modification coefficient at the middle support, *M* is the elastic calculated moment (*M* = 3 *FL*/32), *M*′ is the measured moment, *F* is the applied load, *F_m_* is the measured force at the middle support, and *l* is the length of one span.

**Figure 13:**
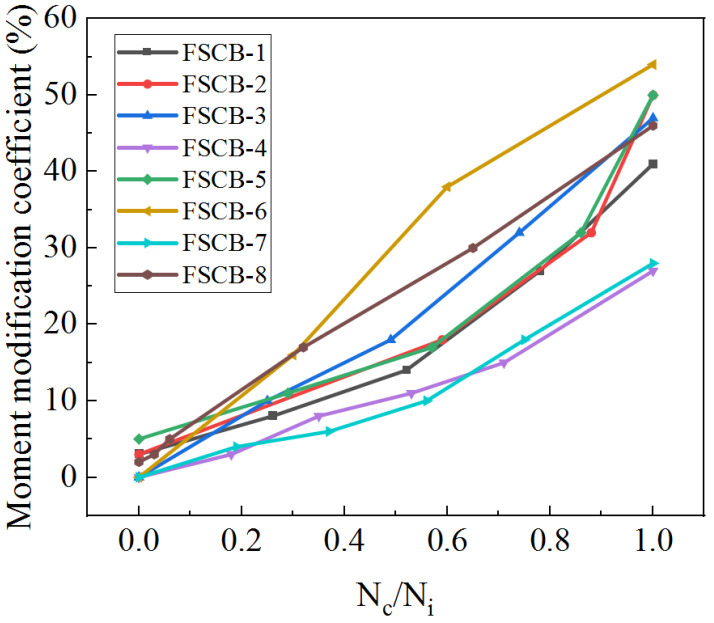
Moment modification coefficient variations.

The moment modification coefficient *β_exp_* at the middle support of FSCB-1 to FSCB-8 versus the fatigue loading process is shown in Figure 13. The moment modification coefficient increases with the increase in the number of fatigue load cycles, and the curves show a quadratic function pattern. When *N_c_/N_i_* = 0, the difference in the moment modification coefficient between FSCB-1 and FSCB-8 is less than 5%. When 0 < *N_c_/N_i_* < 0.5, the moment modification coefficient increases slowly. At this stage, the moment modification coefficients of FSCB-1 to FSCB-8 increase by approximately 11%, 13%, 18%, 10%, 11%, 31%, 9%, and 22%, respectively. When 0.5 < *N_c_/N_i_* < 1, the moment modification coefficients increase faster, and the moment modification coefficients of FSCB-1 to FSCB-8 increase by approximately 27%, 34%, 28%, 16%, 34%, 23%, 19%, and 22%, respectively.

Figure 13 indicates that the growth of the internal force redistribution coincides with the stiffness degradation curve, suggesting that plastic hinges fully develop towards the end of the fatigue loading process. According to the findings in [24], adding reinforcement can effectively restrict concrete cracking in the negative moment zone, reducing the stiffness degradation and internal force redistribution in the entire beam. These results are consistent with the findings for FSCB-4 and FSCB-5 presented in this study. Therefore, stiffness degradation is a critical parameter for calculating steel–concrete continuous composite box beams. As it can be difficult to determine support forces from the existing literature, this study serves as a valuable reference for investigating internal force redistribution in continuous composite box beams throughout the entire fatigue process.

To take the effect of cumulative fatigue damage into account, a quadratic function model is proposed to express the law of the test data, which can be simplified as shown in Equation (5):

*β_n_* = (*β_u_* − *β_s_*) × [*a* × (*N_c_*/*N_i_*)^2^ + *b* × (*N_c_*/*N_i_*)] + *β_s_* (5)

where *a* and *b* are the fatigue effect coefficients, *β_n_* is the moment modification coefficient when *N_c_/N_i_* = *n*, *β_u_* is the moment modification coefficient when *N_c_/N_i_* = 1, and *β_s_* is the moment modification coefficient when *N_c_/N_i_* = 0.

**Figure 14:**
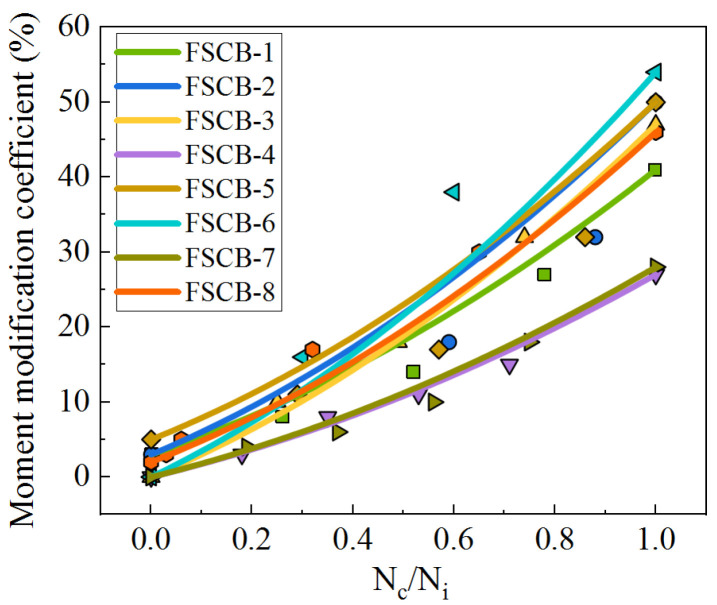
Fitting curves of moment modification coefficient.

By substituting the relevant data in this study into Equation (4), values of *a* = 0.4 and *b* = 0.6 were obtained. The fitting curves are shown in Figure 14. According to the test data, the determination coefficients (R^2^) of all models are above 0.86, as shown in Table 7. This indicates that although the moment modification coefficient is influenced by various factors—such as the amplitude of the fatigue load, reinforcement ratio, stirrup ratio, and stud layout—Equation (5) provides a reasonably accurate fit for all specimens with different parameters.

### 4.2. Internal Force Redistribution at the Middle Support When Approaching Fatigue Failure

**Figure 15:**
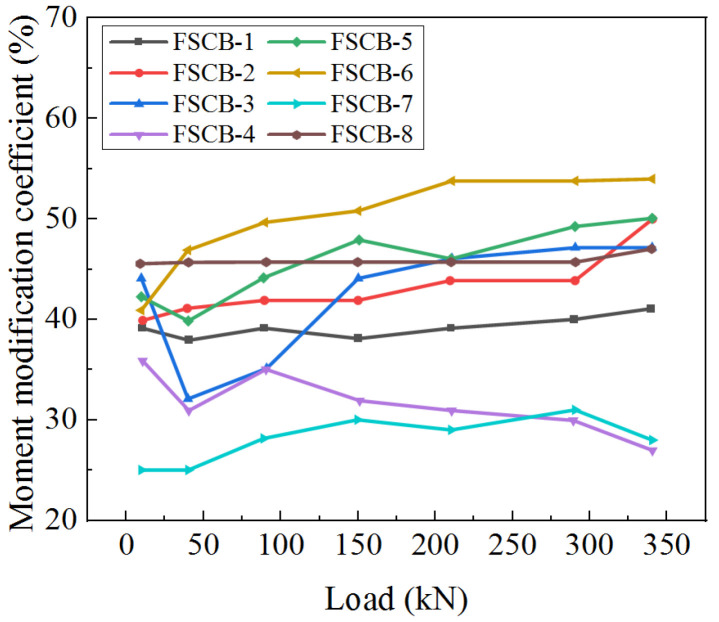
Relation of moment modification coefficient vs. load curves.

Figure 15 shows the moment modification coefficient–load curves for FSCB-1 to FSCB-8 when approaching fatigue failure. The curves indicate that when approaching fatigue failure, a significant moment modification occurred under small loads, but the change in the moment modification coefficient was small with increasing static load. For example, the moment modification coefficient for FSCB-1 was 39% at a load of 10 kN and increased by only 2% to 41% at a load of 340 kN. These results suggest that the plastic hinge in the negative moment zone had fully rotated towards the end of the fatigue loading process. Therefore, for steel–concrete continuous composite box beams approaching fatigue failure, accumulated fatigue damage is the primary factor causing internal force redistribution, rather than the static load.

**Figure 16:**
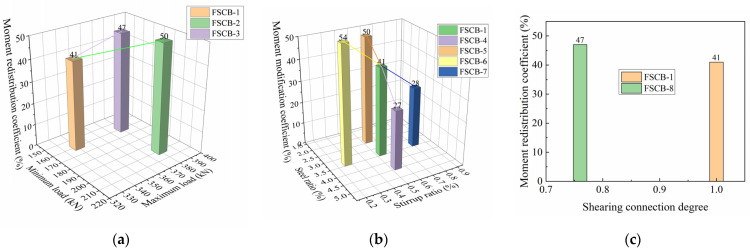
The differences in the moment modification coefficient among specimens: (**a**) relation of moment modification coefficient vs. fatigue load amplitude; (**b**) relation of moment modification coefficient vs. reinforcement ratio and stirrup ratio; (**c**) relation of moment modification coefficient vs. shear connection degree.

The difference in the ultimate moment modification coefficient from FSCB-1 to FSCB-8 is shown in Figure 16. The moment modification coefficient for FSCB-1 to FSCB-8 under 340 kN varied from 27% to 54%. Based on the comparison of each parameter, the following conclusions can be drawn: (1) Figure 16a shows that when the upper and lower load limits increased from 160 kN to 200 kN and from 340 kN to 380 kN, respectively, the moment modification coefficient increased by 9%; when only the upper limit increased from 160 kN to 200 kN, the moment modification coefficient increased by 6%. (2) Figure 16b indicates that when the reinforcement ratio increased from 3.38% to 4.40% or decreased from 3.38% to 2.37%, the moment modification coefficient decreased by 14% or increased by 9%, respectively; when the stirrup ratio increased from 0.54% to 0.81% or decreased from 0.54% to 0.27%, the moment modification coefficient decreased by 13% or increased by 13%, respectively. (3) Figure 16c shows that reducing the shear connection degree from 100% to 76% increased the moment modification coefficient by 5%. This indicates that the moment modification coefficient caused by fatigue load decreases with increasing stirrup ratio, reinforcement ratio, and shear connection degree, and increases with increasing load amplitude and load limit. Among the three comparison groups, the change in the moment modification coefficient with different reinforcement ratios and stirrup ratios is significant (13%), implying that the reinforcement ratio and stirrup ratio are important factors that control the moment modification coefficient under fatigue load.

Based on the plastic hinge rotation theory, Sun [27] obtained the following equations for calculating the moment modification coefficient:

*θ* = Δ*M* × *l* × [2 × (1 − *m*)^3^ + 3 × *m* × *n* × (1 − *m*) × (2 − *n*) + *m*^2^ × *n* × (3 − *m*)]/(3 × *B*) (6)

*θ_u_* = (*φ_u_ − φ_s_*) × *l_p_* (7)

*θ* = *θ_u_* (8)

*β_u,cal_* =Δ*M*/(Δ*M* + *M_u_*) (9)

where *θ* is the angle displacement caused by the modified moment Δ*M*, *l* is the length of one span, *m* is the ratio of the length of the negative moment zone to the length of one span, *n* is the ratio of the stiffness at the positive moment to the stiffness at the negative moment, *B* is the stiffness at the positive moment, *θ_u_* is the ultimate plastic angle displacement of the plastic hinge at the middle support, *φ_u_* is the ultimate curvature when the bottom of the steel box yields, *φ_y_* is the curvature when the reinforcement yields, *l_p_* is the length of the negative moment zone, *M_u_* is the ultimate bending moment at the negative moment, and *β_u,cal_* is the calculated moment modification coefficient.

According to Equations (6)–(9), the moment modification coefficients for FSCB-0 to FSCB-8 and the specimens in references [24,26] were calculated as they approached the ultimate state. The *β_u,cal_* and *β_u,exp_* of each specimen are shown in Table 8. It can be seen from the results that the *β_u,cal_* values of the specimens under static loads are in good agreement with the *β_u,exp_* values. However, for the specimens under fatigue loads, the calculated values are in good agreement with the experimental values for FSCB-1, FSCB-4, FSCB-5, and FSCB-8, while there is some deviation for FSCB-2, FSCB-3, FSCB-6, and FSCB-7. This discrepancy is due to the fact that the stiffness calculation takes into account the influence of the reinforcement ratio and the shear connection degree, but neglects the effects of the fatigue amplitude and stirrup ratio on the stiffness of the negative bending moment zone. In addition, most *β_u,cal_* values under fatigue failure are lower than the *β_u,exp_* values for the specimens under fatigue loads. This is because after a period of fatigue loading, the neutral axis moved downward, so the *M_u_* of specimens was overestimated and the *θ_u_* was underestimated. Hence, it is necessary to take the load amplitude and stirrup ratio into consideration in the calculation of fatigue moment modification.

Overall, the *β_u,cal_* of FSCB-1 to FSCB-8 was within 20% of the *β_u,exp_*. This indicates that Equations (6)–(9) can be applied to steel–concrete composite beams under fatigue loads, but it is necessary to consider the effects of the load amplitude and stirrup ratio on the stiffness and length of the negative moment zone to improve the calculation accuracy.

## 5. Conclusions

To investigate the internal force redistribution in the negative moment zone of steel–concrete continuous composite box beams under fatigue loading, static and fatigue tests were performed on nine specimens. The specific research conclusions are as follows:

1. Under fatigue loading, the failure of steel–concrete composite continuous box beams exhibits a three-stage characteristic, and the fatigue failure mode is the fracture of the upper flange of the steel beam in the negative moment zone.
2. The degradation of the residual stiffness shows an “S-shape pattern”. In the fatigue loading process, the stiffness of the concrete–steel composite box beams decreased more slowly with higher reinforcement ratio or stirrup ratio. When approaching fatigue failure, the residual stiffness of FSCB-1 to FSCB-8 reached 17% to 39%.
3. Compared with the load amplitude or shear connection, the reinforcement ratio and stirrup ratio are significant factors that control the moment modification coefficient under fatigue load. When approaching fatigue failure, an increase of 1.0% in the reinforcement ratio or 0.27% in the stirrup ratio results in a reduction of 13% in the moment modification coefficient.
4. The moment modification coefficient increases with an increase in the number of fatigue load cycles, and the curves show a quadratic function pattern. Based on the test results, this paper proposes a model to calculate the moment modification coefficient for steel–concrete continuous composite box beams during the fatigue process. The model showed good agreement with the test results for all specimens.
5. The findings of this paper verify that the plastic hinge rotation theory is applicable for steel–concrete continuous composite box beams under fatigue load, by comparing the calculated results with the experimental results. However, it is necessary to consider the effects of the load amplitude and stirrup ratio on the stiffness and length of the negative moment zone to improve the calculation accuracy.

## Figures and Tables

**Table 1:** Details of the specimens.

Specimen	Load Amplitude/kN	Shear Connection Degree	Layout of Stud/mm	Space of Stud/mm	Reinforcement Ratio	Stirrup Ratio
FSCB-0	/	1	Φ13 × 116	100	3.38%	0.54%
FSCB-1	160–340	1	Φ13 × 116	100	3.38%	0.54%
FSCB-2	200–380	1	Φ13 × 116	100	3.38%	0.54%
FSCB-3	160–380	1	Φ13 × 116	100	3.38%	0.54%
FSCB-4	160–340	1	Φ13 × 116	100	4.40%	0.54%
FSCB-5	160–340	1	Φ13 × 116	100	2.37%	0.54%
FSCB-6	160–340	1	Φ13 × 116	100	3.38%	0.27%
FSCB-7	160–340	1	Φ13 × 116	100	3.38%	0.81%
FSCB-8	160–340	0.76	Φ13 × 88	130	3.38%	0.54%

Note: The load amplitude at a single loading point is half of the load amplitude in this table.

**Table 2:** Mechanical properties of concrete.

Cube Compressive Strength/MPa	Axial Compressive Strength/MPa	Curing Days/d	Elastic Modulus/MPa
35.5	23.4	28	3.12 × 10^4^

**Table 3:** Mechanical properties of steel.

Type of Material	Thickness or Diameter/mm	Yield Strength/MPa	Ultimate Strength/MPa	Elastic Modulus/MPa
Flange, web	8	324	499	2.02 × 10^5^
Stiffening rib	6	321	480	2.01 × 10^5^
Stirrup	6	320	485	1.95 × 10^5^
Reinforcement steel	14	453	605	2.02 × 10^5^
Studs	13	330	440	2.02 × 10^5^

**Table 4:** The development of concrete cracks (unit: mm).

Specimen	Width of Concrete Cracks					Average Spacing	Length of Negative Moment Zone
	*N_c_* = 0	*N_c_* = 5 × 10^5^	*N_c_* = 1 × 10^6^	*N_c_* = 2 × 10^6^	Final Static Test		
FSCB-1	0.06	0.10	0.15	0.35	0.75	100	1150
FSCB-2	0.08	0.20	-	-	0.90	80	1320
FSCB-3	0.08	0.10	0.14	0.30	0.87	95	1050
FSCB-4	0.04	0.05	0.08	0.15	0.30	90	600
FSCB-5	0.08	0.12	0.28	-	1.07	70	1450
FSCB-6	0.07	0.15	0.20	-	0.85	65	1500
FSCB-7	0.06	0.08	0.13	0.18	0.60	75	830
FSCB-8	0.06	0.11	0.16	-	0.80	95	1200

**Table 5:** Test results of FSCB-1 to FSCB-8.

Specimen	Fatigue Life	Residual Load Capacity/kN	Damage Characteristic	Fatigue Cycles
FSCB-1	1.92 × 10^6^	440	Crack of concrete slab	1.00 × 10^6^
			Slip of steel–concrete interface	1.30 × 10^6^
			Fracture of steel box	1.92 × 10^6^
FSCB-2	8.50 × 10^5^	400	Crack of concrete slab	5.00 × 10^4^
			Slip of steel–concrete interface	5.00 × 10^5^
			Fracture of steel box	8.50 × 10^5^
FSCB-3	2.04 × 10^6^	410	Crack of concrete slab	1.00 × 10^6^
			Slip of steel–concrete interface	1.50 × 10^6^
			Fracture of steel box	2.04 × 10^6^
FSCB-4	2.83 × 10^6^	480	Crack of concrete slab	2.00 × 10^6^
			Slip of steel–concrete interface	2.50 × 10^6^
			Fracture of steel box	2.83 × 10^6^
FSCB-5	1.75 × 10^6^	400	Crack of concrete slab	1.00 × 10^5^
			Slip of steel–concrete interface	1.50 × 10^6^
			Fracture of steel box	1.75 × 10^6^
FSCB-6	1.68 × 10^6^	340	Crack of concrete slab	1.00 × 10^6^
			Slip of steel–concrete interface	1.50 × 10^6^
			Fracture of steel box	1.68 × 10^6^
FSCB-7	2.65 × 10^6^	460	Crack of concrete slab	2.00 × 10^6^
			Slip of steel–concrete interface	2.50 × 10^6^
			Fracture of steel box	2.65 × 10^6^
FSCB-8	1.55 × 10^6^	400	Crack of concrete slab	1.00 × 10^5^
			Slip of steel–concrete interface	5.00 × 10^5^
			Fracture of steel box	1.55 × 10^6^

**Table 6:** Measured force at the middle support during the fatigue load process (unit: kN).

Specimen	Fatigue Load Cycles						
	0	5 × 10^5^	1 × 10^6^	1.5 × 10^6^	2 × 10^6^	2.5 × 10^6^	Fatigue Failure
FSCB-1	234.33	230.69	226.67	218.40	208.94	/	234.33
FSCB-2	234.06	224.08	/	/	/	/	202.66
FSCB-3	235.95	229.36	224.08	214.85	205.81	/	204.95
FSCB-4	236.76	234.21	230.85	228.72	225.86	220.95	218.47
FSCB-5	232.39	228.62	224.48	214.57	/	/	202.92
FSCB-6	238.62	225.68	210.79	205.55	/	/	200.34
FSCB-7	235.63	233.60	231.77	229.18	224.02	218.94	217.28
FSCB-8	234.90	225.07	215.97	206.93	/	/	205.81

**Table 7:** Fitting accuracy of Equation (4).

Specimen	R^2^	Specimen	R^2^
FSCB-1	0.96	FSCB-5	0.86
FSCB-2	0.87	FSCB-6	0.92
FSCB-3	0.99	FSCB-7	0.97
FSCB-4	0.99	FSCB-8	0.98

**Table 8:** Calculated and experimental values of moment modification coefficient.

Specimen	*l_p_/l*	*β_u,cal_*	*β_u,exp_*	*β_u,cal_/β_u,exp_*	Load Type
FSCB-1	0.19	39%	41%	95%	Fatigue load
FSCB-2	0.22	41%	50%	82%	
FSCB-3	0.18	39%	47%	83%	
FSCB-4	0.10	25%	27%	93%	
FSCB-5	0.24	51%	50%	102%	
FSCB-6	0.25	44%	54%	81%	
FSCB-7	0.14	31%	28%	110%	
FSCB-8	0.21	43%	46%	93%	
FSCB-0	0.14	31%	28%	110%	Static load
PC1 [22]	0.13	59%	57%	104%	
PC2 [22]	0.10	49%	45.9%	107%	
PCCB-32 [24]	0.15	62%	58%	107%	
PCCB-33 [24]	0.15	62%	59%	105%	
CCB-34 [24]	0.11	39%	37%	105%	

## Data Availability

All of the data are available within the manuscript.
